# Tuberculosis Disease during Pregnancy and Treatment Outcomes in HIV-Infected and Uninfected Women at a Referral Hospital in Cape Town

**DOI:** 10.1371/journal.pone.0164249

**Published:** 2016-11-03

**Authors:** Adrie Bekker, Hendrik S. Schaaf, Heather R. Draper, Magdalena Kriel, Anneke C. Hesseling

**Affiliations:** 1 Desmond Tutu TB Centre, Department of Paediatrics and Child Health, Faculty of Medicine and Health Sciences, Stellenbosch University, Cape Town, South Africa; 2 DST/NRF Centre of Excellence for Biomedical Tuberculosis Research/Medical Research Council (MRC) Centre for Molecular and Cellular Biology, Division of Molecular Biology and Human Genetics, Faculty of Medicine and Health Sciences, Stellenbosch University, Cape Town, South Africa; University of Cape Town, SOUTH AFRICA

## Abstract

**Background:**

Tuberculosis during pregnancy and treatment outcomes are poorly defined in high prevalence tuberculosis and HIV settings.

**Methods:**

A prospective cohort study of pregnant and postpartum women identified to be routinely on antituberculosis treatment was conducted at Tygerberg Hospital, Cape Town, South Africa, from January 2011 through December 2011. Maternal tuberculosis disease spectrum and tuberculosis-exposed newborns were characterized by maternal HIV status. Maternal tuberculosis treatment outcomes were documented and a multivariable regression model identified predictors of unfavourable tuberculosis treatment outcomes. Infant outcomes were also described.

**Results:**

Seventy-four women with tuberculosis, 53 (72%) HIV-infected, were consecutively enrolled; 35 (47%) were diagnosed at delivery or postpartum and 22 (30%) of women reported previous antituberculosis treatment. HIV-infected women were 5.67 times more likely to have extrapulmonary tuberculosis (95% CI 1.18–27.25, p = 0.03). All 5 maternal deaths were amongst HIV-infected women. Birth outcomes were available for 75 newborns (2 sets of twins, missing data for 1 stillbirth). Of the 75 newborns, 49 (65%) were premature and 44 (59%) were low birth weight (LBW; <2500 grams). All 6 infants who died and the 4 stillbirths were born to HIV-infected women. Unfavourable tuberculosis treatment outcomes were documented in 33/74 (45%) women. Unfavourable maternal tuberculosis outcome was associated with delivery of LBW infants (OR 3.83; 95% CI 1.40–10.53, p = 0.009).

**Conclusions:**

A large number of pregnant women with tuberculosis presented at a provincial referral hospital. All maternal and infant deaths occurred in HIV-infected women and their newborns. Maternal tuberculosis treatment outcomes were poor.

## Introduction

Tuberculosis (TB) during pregnancy and maternal TB treatment outcomes are poorly defined in settings with high burden of TB and human immunodeficiency virus (HIV). TB and HIV infection are both associated with increased morbidity and mortality in pregnant women and their infants [1–4]. The World Health Organization (WHO) estimated that there were 9.6 million new TB cases globally in 2014, of which 1.2 million (12%) were HIV-infected, 74% from the African region [5]. Of these incident TB cases, 3.2 million occurred in women, with 480, 000 estimated TB deaths [5].

It is unclear how many of these women were pregnant, as most countries do not report the pregnancy status of female TB cases. Women of reproductive age are disproportionately affected by TB in high HIV prevalence settings [6–7]. HIV-infected women are at increased risk of TB with a prevalence of TB in pregnant women ranging from 1–11%, compared to 0.06–0.53% in HIV-uninfected women [8]. Immunological changes during pregnancy may predispose to the susceptibility of new infection and the activation of latent TB infection [8]. Whether this increased risk of TB in HIV-infected women is further increased by being pregnant, remains unknown. In South Africa, where 61% of all reported TB cases were HIV-infected in 2014 [5], non-pregnancy related infections (mainly deaths in HIV-infected women complicated by TB and pneumonia) are the single most common cause of maternal mortality, accounting for more than 35% of all maternal deaths [9]. TB in pregnant women also adversely impacts on perinatal and infant outcomes. A two-fold risk of delivering premature (<37 weeks gestational age) and low birth weight (LBW; <2500 grams) infants, and a six-fold increase in perinatal deaths have been reported in women with TB [10]. Limited data are available for TB treatment outcomes in pregnant women with TB, despite both pregnancy and TB linked to poor outcomes [11].

The aim of this study was to describe the clinical presentation of TB in HIV-infected and HIV-uninfected pregnant women and their perinatal outcomes at a large provincial referral hospital in Cape Town. Maternal TB treatment outcomes were assessed at the end of antituberculosis treatment. Predictors of unfavourable TB treatment outcomes in pregnant women were identified and reported.

## Methods

### Study design and setting

We conducted a prospective cohort study in HIV-infected and HIV-uninfected pregnant women with TB routinely admitted to the obstetric services at Tygerberg Hospital (TBH), Cape Town, South Africa, from January 2011 through December 2011. TBH is a large secondary and tertiary provincial referral hospital in the Western Cape Province serving approximately one-third of the provincial population. During 2011, 8471 women were admitted to the obstetric services at TBH, which managed 5864 high-risk deliveries. Obstetric service admissions included women with antepartum (prior to delivery) complications, women in labour (intrapartum), and women with postpartum complications (until 6 weeks post delivery). LBW infants constituted 2179 (37%) of all live births during this period [12]. In 2009, Cape Town had a mid-year population of 3,443,010 (3,241,508 HIV-uninfected and 201,502 HIV-infected individuals), and 29,478 newly notified TB cases were recorded in the electronic TB registry (ETR.net) [13]. Pregnant and postpartum women identified to be on routine antituberculosis treatment at TBH were consecutively enrolled and baseline information was obtained from mothers and their newborns.

### Pregnant women with tuberculosis

Antenatal TB screening was not routinely recommended or implemented by the South African National TB programme (NTP) during the study period, despite the fact that all HIV-infected persons were recommended to have had routine TB screening [14]. TB screening in pregnant women at TBH consisted of identifying any of the following symptoms: presence of chronic cough (>2 weeks), night sweats, fever, and loss of weight or failure to gain weight during pregnancy. If any of these were present, investigations were performed, including sputum/other samples for microscopy for acid-fast bacilli (AFB) and mycobacterial culture, and shielded chest radiography in the case of suspected pulmonary TB (PTB) [14]. For women with suspected extrapulmonary TB (EPTB), directed special investigations were performed as clinically indicated. The type of TB was classified according to the anatomical site of disease using standard WHO definitions: PTB only involved the lung parenchyma or the tracheobronchial tree; EPTB involved organs other than the lungs, i.e. pleura, lymph nodes, pericardium, bones, meninges or positive blood cultures for *Mycobacterium tuberculosis*; both PTB and EPTB involved a PTB case complicated by pleural effusion [15]. For the purpose of the study, severe EPTB manifestations were defined as EPTB not isolated to only peripheral lymph nodes or pleural effusions, i.e. TB meningitis, *M*. *tuberculosis* positive on blood culture, abdominal TB, TB spine and TB pericarditis. Standard WHO TB case definitions were applied: both a bacteriologically confirmed TB case (a biological specimen that is positive by smear, microscopy, culture or Xpert MTB/RIF) or a clinically diagnosis of TB (no bacteriological confirmation but diagnosed as TB disease with a decision to treat by a routine clinician) were classified as maternal TB cases [15]. The standard antituberculosis treatment regimen for drug-susceptible TB, also in pregnant women, included a 2-month intensive phase of isoniazid (INH), rifampicin (RMP), pyrazinamide (PZA) and ethambutol, followed by a 4-month continuation phase of INH and RMP [14]. Antituberculosis treatment was individualised based on WHO treatment guidance. Drug-resistant antituberculosis treatment regimens consisted of at least four effective drugs and the total antituberculosis treatment duration was maintained for a minimum of 18 months [16]. No injectable agents were used in pregnant women with drug-resistant TB due to the risk of foetal toxicity [14].

### TB-exposed newborns

If any mother with TB was judged to pose a risk of transmitting *M*. *tuberculosis* to her newborn (i.e. mother on antituberculosis treatment for < 2 months or not yet sputum smear or culture converted), infants were investigated for TB disease. Infant screening included clinical examination, gastric aspirates/other sample for culture for *M*. *tuberculosis*, chest radiography and other imaging/investigations as clinically indicated. Antituberculosis treatment was started in infants if TB was suspected or confirmed. In disease-free infants, where the mother might still be infectious, 10 mg/kg/day of isoniazid preventive therapy (IPT) for six months was given in infants born to drug-susceptible TB mothers, while infants born to mothers with drug-resistant TB received tailored preventive therapy regimen according to the mother’s drug susceptibility test result. Infants born to mothers with TB who were no longer judged to be infectious did not receive antituberculosis treatment, but were closely followed as part of routine care. Infant TB outcomes were available for TB-exposed infants at 6 months of age in a subset of infants enrolled on a complementary study of IPT delivery [17].

### HIV infection in women and newborns

Maternal HIV testing included two confirmatory enzyme-linked immunosorbent assays (ELISA) for HIV antibodies, routinely performed in all pregnant women with an unknown HIV status upon hospital admission. The prevention of mother-to-child HIV transmission (PMTCT) policy at the time included lifelong combination antiretroviral therapy (cART) for all HIV-infected pregnant women with a CD_4_ count ≤350 cells/mm^3^ and limited ART from 14 weeks onwards during pregnancy for HIV-infected women with CD_4_ count >350 cells/mm^3^ [18]. HIV-infected pregnant women on cART received the following drugs, tenofovir (TDF), lamivudine (3TC)/emtracitabine (FTC) plus nevirapine (NVP). Limited ART regimens included antepartum (just before delivery) zidovudine (AZT), intrapartum (during delivery) NVP and AZT, and a single dose of TDF and FTC postpartum. Daily NVP for 6 weeks was administered as newborn preventive therapy for HIV, or daily NVP was given until discontinuation of breastfeeding for mothers not on cART. HIV polymerase chain reaction (PCR) testing was routinely performed at 6 weeks of age in HIV-exposed infants and earlier if clinically indicated. All HIV-infected infants were fast-tracked for cART initiation [19]. HIV PCR results for infants were extracted from the provincial laboratory database 6 months post-delivery.

### Maternal TB treatment outcomes

Maternal TB treatment outcomes were assessed at the end of antituberculosis treatment, which was routinely administered by TB services. Facility-based TB treatment clinic treatment registers (from local clinics) were reviewed and verified against the national electronic TB registry. Standard WHO TB treatment outcome definitions were applied to classify maternal TB disease outcomes: cured, treatment completed, treatment failure, lost to follow-up or death [15]. Cured or treatment completion was considered favourable TB treatment outcomes, while treatment failure, lost to follow-up (LTFU) or death were classified as unfavourable TB treatment outcomes. Maternal mortality was defined using the International Classification of Diseases revised version 10, which included late maternal deaths between 42 days and one-year post abortion, miscarriage or delivery [20].

### Data collection

Written informed consent was obtained from eligible women with TB. Baseline information was extracted from maternal and infant hospital folders using a standard data collection tool. Maternal data included age, ethnicity (black versus mixed race), haemoglobin at admission, previous antituberculosis treatment, the presence of patient-reported TB symptoms (cough of any duration, night sweats, perceived weight loss, chest pain or shortness of breath, fever, tiredness or malaise), the timing of TB diagnosis (ante- versus intra- or postpartum), type of TB (PTB, EPTB or both), TB treatment regimen, and microbiology results including AFB smear-microscopy, culture, and drug susceptibility testing where available. All chest radiographs were read by two independent readers, using the modified Timika radiology score [21], which estimates the extent of active PTB seen on a posteroanterior chest radiograph according to percentage of lung involvement and the presence/absence of cavities [22]. In HIV-infected women CD_4_ count during pregnancy and ART delivery were documented. Perinatal outcomes included gestational age, birth weight, birth type (singleton versus twin status), and death (stillbirth defined as death of a foetus after 28 weeks gestation). All antituberculosis treatment decisions in newborns and deaths prior to hospital discharge were recorded. Maternal TB treatment outcomes were assessed at antituberculosis treatment completion.

### Data analysis

Maternal TB and infant characteristics, including infant outcomes, were evaluated by maternal HIV status using summary statistics, odds ratios (OR) and 95% confidence intervals (CI). Bivariate associations between categorical variables were evaluated using the Chi-square or Fisher’s exact tests and continuous variables by t-test or Wilcoxon sign-rank-sum test for normally and non-normally distributed data, respectively. A p-value <0.05 was used to determine statistical significance. In addition, a multivariable regression model was used to identify whether any covariates were associated with unfavourable maternal TB treatment outcome. Any covariate with a univariable logistic regression p-value <0.1 was included in the final model. Maternal HIV status was considered clinically relevant and was included in the final model regardless of its univariable p-value.

The study was approved by the Stellenbosch University Health Research Ethics Committee (N10/08/279), and the Ethics Advisory Group of the International Union Against Tuberculosis and Lung Disease (944/10), Paris, France.

## Results

### Characteristics of pregnancy-associated TB

**Fig 1:**
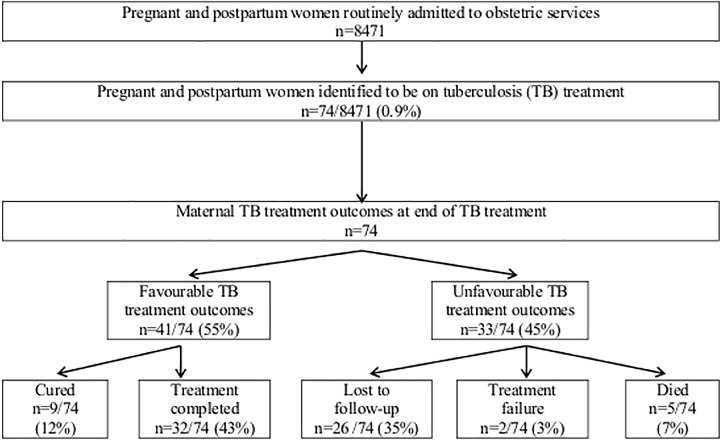
Pregnant and postpartum women on tuberculosis treatment and their outcomes (n = 74).

Seventy-four pregnant and postpartum women were identified to be on antituberculosis treatment at TBH (Fig 1). Sixty-one (82%) of 74 women delivered at TBH and 13/74 (18%) women were transferred in post-delivery. Of the 74 women with TB, 39 (53%) were started on antituberculosis treatment prior to the delivery (3.6 months median duration of TB treatment) and 35 (47%) women were initiated on TB treatment upon hospital admission for delivery or within 6 weeks post-delivery. On admission to the obstetric services, symptoms suggestive of TB were documented in 31/74 (42%) women: 28/31 (90%) reported cough of any duration, 11/31 (35%) night sweats, 11/31 (35%) perceived weight loss, 11/31 (35%) chest pain or shortness of breath, 6/31 (19%) reported fever and 2/31 (6%) complained of tiredness and malaise.

Table 1 describes the maternal TB characteristics by HIV status. Fifty-three (72%) of 74 women on antituberculosis treatment were HIV-infected (median CD_4_ count 155 cells/mm^3^; range 11–565 cells/mm^3^); 29/53 (55%) were on cART at time of delivery (median time on cART 2 months; IQR: 1–5 months), 5/53 (9%) on PMTCT, 10/53 (19%) received no ART, and 9/53 (17%) had no documentation of ART. Age, ethnicity and EPTB only were associated with maternal HIV status. All severe EPTB manifestations occurred in HIV-infected women. Six (14%) of 42 women in whom TB was bacteriologically confirmed had drug-resistant TB; 4 were HIV-infected and 4 overall had unfavourable treatment outcome (Table 2).

**Table 1:** Maternal tuberculosis characteristics by HIV status (n = 74).

	All pregnant women	Maternal HIV status		OR (95% CI)	p-value
		HIV-infected (n = 53)	HIV-uninfected (n = 21)		
**Age in years, mean (SD)**	29.8 (6)	31.1 (6)	26.7 (6)	1.16 (1.04–1.30)	0.006
**Black ethnicity (%)** ^**1**^	48 (65)	42 (80)	6 (29)	9.55 (3.00–30.34)	<0.001
**Haemoglobin in g/dL, mean (SD) [n = 70]**	9.8 (1.7)	9.6 (1.8)	10.1 (1.5)	0.86 (0.64–1.18)	0.352
**Previous TB treatment (%)**	22 (30)	16 (30)	6 (29)	1.08 (0.35–3.29)	0.891
**Timing of TB diagnosis**					
Antepartum (%)	39 (53)	27 (51)	12 (57)	-Ref-	
Intra- or postpartum (%)	35 (47)	26 (49)	9 (43)	1.28 (0.46–3.55)	0.630
**TB case definitions**					
Bacteriologically confirmed (%)	50 (68)	33 (62)	17 (80)	0.39 (0.11–1.32)	0.129
Clinically diagnosed (%)	24 (32)	20 (38)	4 (20)	Ref	
**TB disease type**					
PTB only (%)	47 (63)	30 (56)	17 (80)	Ref	
EPTB only (%)	22 (30)	20 (38)	2 (10)	5.67 (1.18–27.25)	0.030
Both PTB and EPTB (%)	5 (7)	3 (6)	2 (10)	0.85 (0.13–5.60)	0.866
**EPTB disease manifestation [n = 27]** ^**2**^					
*Severe EPTB*					
TB meningitis (%)	8 (29)	8 (15)	0 (0)		
Blood culture positive for *M*. *tuberculosis* (%)	1 (4)	1 (2)	0 (0)		
Abdominal TB (%)	1 (4)	1 (2)	0 (0)		
TB spine (%)	1 (4)	1 (2)	0 (0)		
TB pericarditis (%)	1 (4)	1 (2)	0 (0)		
Non severe EPTB (%)					
Peripheral lymph nodes (%)	2 (7)	2 (4)	0 (0)		
Pleural effusions (%)	13 (48)	9 (17)	4 (19)		
**Microbiological results**					
Smear positive (%) [n = 73]^3^	28 (38)	18 (34)	10 (50)	0.44 (0.16–1.24)	0.121
Culture positive (%)	42 (57)	28 (53)	14 (67)	0.56 (0.19–1.61)	0.282
**Chest radiographic features [n = 52]** ^**4**^	52 (70)	39 (74)	13 (62)		
Percentage affected lungs, median (IQR)	14 (8–32)	14 (8–33)	14 (8–28)	0.99 (0.96–1.02)	0.382
Presence of cavities (%)	9 (17)	6 (15)	3 (23)	0.61 (0.13–2.87)	0.528
Final score, median (IQR)	15 (8–38)	19 (8–38)	14 (8–28)	0.99 (0.97–1.01)	0.346
**TB treatment regimen**					
First-line TB treatment (%)	68 (92)	49 (93)	19 (91)	1.29 (0.22–7.63)	0.779
Second-line TB treatment (%)	6 (8)	4 (7)	2 (9)		
**Maternal mortality (%)**	5 (7)	5 (9)	0 (0.0)	—	0.313

OR, odds ratio; SD, standard deviation; TB, tuberculosis; Ref, reference; PTB, pulmonary TB; EPTB, extrapulmonary TB; IQR, interquartile range.

^1^ Women were of black ethnicity or mixed race

^2^ *M*. *tuberculosis* was cultured in 15/27 (56%) EPTB women; on cerebrospinal fluid in 3, on bone biospy in 1, on pericardial fluid in 1, on blood culture in 1, on lymph node tissue in 3 (1 abdominal TB and 2 peripheral lymphadenopathy), and on pleural fluid in 6.

^3^ Xpert MTB/RIF was positive and culture positive for *M*. *tuberculosis* in the sputum of 1 women. The positive Xpert MTB/RIF result was excluded from the smear positive analysis.

^4^ Chest radiographic features were only available for 52 pregnant women with any TB disease type.

**Table 2:** Characteristics and outcomes of pregnant women with confirmed drug-resistant tuberculosis (n = 6).

Maternal characteristics			Outcomes	
TB presentation	TB treatment regimen	HIV features	Perinatal and infant ^1^	Maternal TB
• Pulmonary TB^2^ • *M*. *tb* (INH and RMP resistant) cultured from sputum within 13 d • 38 y, • Mixed race	• 3 m of OFX,ETO,PZA,EMB,TRD prior to delivery; • 9 m interruption post- delivery, and presented again with *M*.*tb* sputum (INH, RMP, ETH and OFX resistant)	• CD_4_ 24 cells/mm^3^ • Previously diagnosed with HIV infection, 3 m prior to delivery (TDF, 3TC, NVP)	▪ Male, ▪ 1940 g, ▪ 38 w GA ▪ Alive at 9 m and completed 6 m of high dose INH, in place of safety. ▪ PCR HIV negative	Unfavourable
				(LTF)
▪ Pulmonary TB^2^ ▪ *M*. *tb* (INH and RMP resistant) cultured from sputum within 22 d ▪ 28 y, ▪ African	▪ 8 m of OFX, ETO, PZA,EMB,TRD and 5 m of TB treatment interruption prior to delivery. ▪ Mom was re-initiated on TB treatment post-delivery—adhered for 3 m, and then re-located	• CD_4_ 410 cells/mm^3^ • Previously diagnosed with HIV infection, but defaulted ART 5 m prior to delivery PMTCT at delivery	▪ Male, ▪ 3100 g, ▪ 40 w GA ▪ Alive at 3 m and completed 3 m of high dose INH, then LTF. ▪ PCR HIV negative	Unfavourable
				(LTF)
• TB meningitis and miliary TB • *M*. *tb* (INH and RMP resistant) cultured from CSF within 18 d, • 29 y, • Mixed race	• 11 days of INH,RMP,PZA,EMB • *M*.*tb* culture results only became available after the mother had died	• CD_4_ 87 cells/mm^3^ • Previously diagnosed with HIV infection, but defaulted ART 3 m prior to delivery	▪ Foetus died *in utero*	Unfavourable
				(died)
• Pulmonary TB ^2^ • *M*. *tb* (INH and RMP resistant) cultured from sputum within 22 d • 30 y, • African	• 18 m of OFX,ETO,PZA,EMB,TRD • 14 m prior to delivery, and 4 m post-delivery	• CD_4_ 154 cells/mm^3^ • Previously diagnosed with HIV infection 14 m prior to delivery (ZDV, 3TC, EFV)	▪ Female, ▪ 2690 g, ▪ 40 w GA ▪ Alive at discharge from hospital. No TB medication indicated ▪ PCR HIV negative	Favourable
				(cured)
• Pulmonary TB • *M*. *tb* (INH and RMP resistant) cultured from sputum within 22 d • 38 y, • Mixed race	• 24 m of OFX,ETO,PZA,EMB,TRD • 9 m prior to delivery, and 15 m post-delivery	• HIV uninfected	▪ Male, ▪ 2920 g, ▪ 38 w GA ▪ Alive at discharged from hospital. ▪ No TB medication indicated	Favourable
				(cured)
Pulmonary TB • s*M*. *tb* (INH, RMP, ETH, OFX and AMK resistant) cultured from sputum within 24 d • 30 y, • African	• 1 m of OFX,ETO,PZA,EMB,TRD • Prior to delivery, followed by 8 m of extensively drug-resistant TB treatment ^3^	• HIV uninfected	▪ Female, ▪ 3625 g, ▪ 40 w GA ▪ Alive at 1 month and completed 1 month of high dose INH—then LTF (re-located with grandmother)	Unfavourable
				(LTF)

TB, tuberculosis; *M*. *tb*, *Mycobacterium tuberculosis*; INH, isoniazid; RMP, rifampicin; d, days; y, years; m, months; OFX, ofloxacin; ETO, ethionamide; PZA, pyrazinamide; EMB, ethambutol; TRD, terizidone; TDF, tenofovir; 3TC, lamivudine; NVP, nevirapine, g, grams; ZDV, zidovudine; EFV, efavirenz; w, weeks; GA, gestational age; ART, antiretroviral therapy; PCR, polymerase chain reaction; PMTCT, Prevention of mother-to-child-HIV transmission; CSF, cerebrospinal fluid

^1^ No fetal abnormalities were detected at birth

^2^ Previously treated for drug-susceptible TB

^3^ Mother was subsequently discharged from TB hospital and lost to follow-up

### Maternal TB treatment outcomes

Forty-one (55%) of 74 women with TB had favourable TB treatment outcomes and 33 (45%) women had unfavourable treatment outcomes (Fig 1). Of the 26/74 (35%) women who were LTFU, 13 were LTFU before entering the clinic TB registry and 13 women were classified as LTFU at the time of TB treatment completion. HIV status was not associated with unfavourable maternal TB treatment outcome (OR 1.1, 95% CI 0.4–3.1, p = 0.85), but all 5 women who died were HIV co-infected. Deaths amongst pregnant women are described in Table 3. All five maternal deaths (3 during hospital delivery admission and 2 after discharge from hospital) were classified as TB-related by routine attending clinicians, but no autopsies were performed.

**Table 3:** Deaths amongst pregnant women with tuberculosis and infant outcomes (n = 5).

Maternal characteristics			Outcomes	
TB presentation	TB treatment regimen duration prior to delivery	HIV features at delivery	Perinatal and infant	Maternal death
▪ Pulmonary TB ▪ Respiratory failure (Sputum negative for *M*. *tb*) ▪ 31 y and African ▪ Hb 11.4 g/dL	▪ 1 w ▪ RMP,INH,PZA and EMB	▪ *Pneumocystis jiroveci* pneumonia ▪ CD_4_ 16 cells/mm^3^. ▪ Newly diagnosed with HIV ▪ No prior cART	▪ Male, 1290 g, 29 weeks GA ▪ Alive at 3 m and on TB preventive therapy ▪ PCR HIV negative	▪ 2 d after delivery
▪ Abdominal TB ▪ *M*. *tb* (INH and RMP susceptible) cultured from lymph node within 6 d. ▪ Adenosine deaminase of 185 IU/L on ascitic fluid ▪ 24 y and African ▪ Hb 9.8 g/dL	▪ 1 d ▪ RMP,INH,PZA and EMB	▪ Kaposi sarcoma ▪ CD_4_ 50 cells/mm^3^ ▪ Diagnosed with HIV 2 m prior to delivery ▪ cART initiated at time of HIVdiagnosis	▪ Female, 2030 g, 35 weeks GA ▪ *M*. *tb* cultured from gastric aspirate (x2). Alive at 6 m and completed TB treatment ▪ PCR HIV negative	▪ 96 d after delivery
▪ Bacteraemia ▪ *M*. *tb* (INH and RMP susceptible) cultured from blood within 28 d ▪ 27 y and African ▪ Hb 7.8 g/dL	▪ Less than 1 m ▪ RMP,INH,PZA and EMB	▪ CD_4_ 102 cells/mm^3^ ▪ Newly diagnosed with HIV ▪ cART inititated < 1 m prior to delivery	▪ Male, 980 g, 30 weeks GA ▪ Died on day of birth—cause of death: prematurity and hyaline membrane disease ▪ PCR HIV negative	▪ On day of delivery
▪ TB meningitis and miliary TB ▪ *M*. *tb* (INH and RMP resistant) cultured from CSF within 18 d ▪ 29 y and of mixed race ▪ Hb 8.2 g/dL	▪ 11 d ▪ RMP,INH,PZA and EMB	▪ CD_4_ 87 cells/mm^3^ ▪ Newly diagnosed with HIV ▪ No prior cART	▪ Foetus died *in utero*	▪ 11 d after admission
▪ TB meningitis ▪ Tuberculoma on CT scan ▪ 27 y and of mixed race ▪ Hb 7.3 g/dL	▪ 1 d ▪ RMP,INH,PZA and EMB	▪ CD4 37 cells/mm^3^ ▪ Newly HIV diagnosed ▪ No prior cART	▪ Male, 1260 g, 30 weeks GA ▪ Alive at 6 m and completed TB treatment ▪ PCR HIV negative	▪ 54 d after delivery

*M*. *tb*, *M*. *tuberculosis*; y, years; RMP, rifampicin; INH, isoniaizd; PZA, pyrazinamide; EMB, ethambutol; cART, combination antiretroviral therapy; g, grams; w, weeks; GA, gestational age; PCR, polymerase chain reaction; d, days; Hb, haemoglobin; m, months; CSF, cerebrospinal fluid; CT, computerized tomography

### Characteristics and outcomes of TB-exposed infants

Table 4 summarizes the characteristics and hospital outcomes of infants by maternal HIV status (n = 76; 2 sets of twins). Gestational age and birth weight was unknown for 1 stillbirth.

**Table 4:** Characteristics and outcome of TB-exposed infants at hospital discharge, by infant HIV exposure status (N = 76).

	All infants	Infant HIV Status		OR (95% CI)	p-value
		HIV-exposed (n = 55)	HIV-unexposed (n = 21)		
**Gestational age in weeks, median (IQR) [n = 75]** ^**1**^	36 (31–38)	36 (31–38)	37 (33–38)	0.99 (0.87–1.13)	0.851
**Premature (%) [n = 75]** ^**1**^	49 (65)	38 (70)	11 (52)	2.16 (0.77–6.09)	0.146
**Birth weight in grams, median (IQR) [n = 75]** ^**1**^	2120 (1386–2920)	2080 (1350–2930)	2230 (1900–2885)	1.0 (1.00–1.00)	0.563
**Low birth weight, <2500 grams (%) [n = 75]** ^**1**^	44 (59)	32 (59)	12 (57)	1.09 (0.39–3.03)	0.867
**Birth type**					
Singletons (%)	72 (95)	51 (93)	21 (100)	—	0.571
Number of twin babies (%)	4 (5)	4 (7)	0 (0)		
**TB treatment decision in live born infants [n = 72]**					
No TB treatment (%)	11 (15)	7 (14)	4 (19)	Ref	
TB preventive therapy (%)	57 (79)	41 (80)	16 (76)	1.46 (0.38–5.69)	0.582
TB treatment (%) ^2^	4 (6)	3 (6)	1 (5)	1.71 (0.13–22.51)	0.682
**Deaths**	10 (13)	10 (18)	0 (0.0)	—	0.054
Stillbirths (%)	4 (5)	4 (7)	0 (0.0)		
Newborn deaths (%) ^3^	6 (8)	6 (11)	0 (0.0)		

OR, odds ratio; CI, confidence interval; IQR, interquartile range; Ref, reference; AFB, acid fast bacilli

^1^ Unknown gestational age and birth weight for one fetus who was still in utero when mother died.

^2^ *M*. *tuberculosis* cultured from 2 gastric aspirates in 1 infant, 1 symptomatic ventilated infant had AFB on tracheal aspirate, 1 infant had miliary TB, and 1 infant had suggestive chest radiography.

^3^ Respiratory distress syndrome in 2, necrotising enterocolitis in 1, presumed nosocomial sepsis in 1, vein of Galen malformation in 1, and duodenal web in 1.

Forty-nine of 75 (65%) infants were premature and 44/75 (59%) infants were LBW. No differences in the gestational age and birth weight were observed between HIV-exposed and HIV-unexposed infants. All ten deaths (4 stillbirths, and 6 newborn deaths) were in infants born to HIV-infected women; the causes of infant deaths are listed in Table 4. Treatment decisions were made for all 72 live-born TB-exposed infants (4 stillbirths); 11 (15%) were given no antituberculosis treatment by routine attending clinicians (3 newborn deaths, 8 born to women judged not to be infectious); 57 (79%) were initiated on IPT (all born to women judged to pose significant *M*.*tuberculosis* transmission risk), and 4 (6%) were started on antituberculosis treatment (3 completed 6 months of antituberculosis treatment and 1 died). *M*.*tuberculosis* was confirmed in 2/72 (3%) TB-exposed infants; both infants had congenital TB and both were born to HIV-infected women.

Of the 57 infants initiated on IPT, outcome data were available in 39 (68%) infants at 6 months following hospital discharge: 24/39 (62%) completed IPT, 13/39 (33%) did not complete IPT, and 2/39 (5%) died (17). HIV PCR tests were performed in 45 (82%) of 55 HIV-exposed newborns; HIV PCR testing was not done in 10 infants (4 stillbirths, 4 newborn deaths, and in 2 newborns who were LTFU). Of the 45 newborns who had HIV testing, 42 (93%) had a negative test result and 3 (7%) were HIV PCR positive. All 3 HIV-infected infants were initiated on cART and were alive and well at six months of age.

### Predictors of unfavourable maternal TB treatment outcomes

In univariable logistic regression, delivery of a LBW infant was associated with unfavourable maternal TB treatment outcome (p = 0.009). Maternal HIV status, maternal age, haemoglobin upon hospital admission, the presence of any EPTB, bacteriologically confirmed tuberculosis, and intra- and postpartum TB diagnosis were not associated with an unfavourable treatment outcome (Table 5). In multivariable regression, adjusting for maternal HIV infection, women delivering LBW infants were 3.83 times more likely to have an unfavourable TB treatment outcome (95% CI 1.40–10.53, p = 0.009), compared to women delivering infants weighing > 2500 grams.

**Table 5:** Predictors of unfavourable maternal tuberculosis treatment outcome (n = 74).

	Univariable OR (95% CI)	p-value	Multivariable OR (95% CI)	p-value
Maternal HIV infection	1.10 (0.40–3.06)	0,850	1.06 (0.36–3.10)	0.921
Low birth weight infant (<2500g)	3.83 (1.40–10.53)	0,009	3.83 (1.40–10.53)	0.009
Any extrapulmonary TB	1.59 (0.61–4.12)	0,342		
Maternal age	0.99 (0.92–1.07)	0,837		
Intra- and postpartum TB diagnosis	1.69 (0.67–4.27)	0,264		
Haemoglobin prior to delivery	0.87 (0.66–1.15)	0,319		
Bacteriologically confirmed TB	1.54 (0.57–4.16)	0,396		

## Discussion

This study represents one of the largest prospective cohorts of HIV-infected and uninfected pregnant and postpartum women with TB, including reporting of maternal TB treatment outcomes. TBH is a provincial referral hospital that manages high-risk and complicated deliveries and serves communities with high burdens of TB and HIV. Of the 74 women with TB, nearly half were only diagnosed at delivery or in the postpartum period, and almost a third of women reported prior tuberculosis treatment. More than two-thirds of women on antituberculosis treatment were HIV-infected, the majority were severely immune suppressed, and many presented with severe manifestations of EPTB. All deaths occurred in HIV-infected women. All stillbirths and newborns who died were born to HIV-infected women. Maternal TB treatment outcomes were poor with unfavourable TB treatment outcomes in 33/74 (45%) of women. Prematurity and LBW status were common amongst infants. LBW deliveries were associated with unfavourable maternal TB treatment outcomes.

The challenges of diagnosing TB during pregnancy may lead to under-recognition of TB in pregnant women. Early TB symptoms are often non-specific [23], and overlap with pregnancy symptoms [11]. Poor performance of TB symptom-screening tools has been noted in two recent studies in HIV-infected pregnant women, reporting a sensitivity of 28% and 54%, respectively [24–25]. A third of women from our cohort reported previous TB treatment, highlighting the possible importance of documentation of previous treatment as a risk factor for maternal TB. Consideration of previous TB may therefore be useful as an additional part of current WHO TB screening algorithms in pregnant women. Furthermore, TB diagnosis in pregnant women was often delayed. Almost half of the women in our cohort were only diagnosed with TB upon hospital admission for labour and up to 6 weeks post-delivery. Delayed TB diagnosis can be explained both by the difficulty in diagnosing TB during pregnancy and the increased risk of TB in the post-partum period. A large epidemiological study from the United Kingdom that was conducted in 192,801 women (a total of 264,136 pregnancies) recently found that early postpartum women were twice as likely to develop TB as non-pregnant women [26]. Delayed TB diagnosis in 47% of our cohort likely contributed to the pronounced maternal morbidity and mortality. Untreated maternal TB carries a significant risk of TB exposure to infants and risk of TB in infants, and led to IPT being initiated in three-quarters of TB-exposed infants.

More than two-thirds (72%) of women with TB were HIV-infected, which is comparable to the 2009 national data with an HIV prevalence of 61% in all notified TB cases [5]. Despite wide access to maternal ART, only 64% ART uptake was documented in pregnant women with TB in our study. Only HIV-infected women had severe EPTB manifestations, including TB meningitis, TB spine, TB pericarditis, abdominal TB and bacteraemia. Increased EPTB manifestations in HIV-infected pregnant women have also been reported in studies of TB during pregnancy (1, 3). Whether this increased risk for EPTB is purely because of immunosuppression from HIV co-infection [27–28], and whether the immunological changes from pregnancy co-contribute, remain uncertain. All five maternal deaths occurred in HIV-infected women and were attributed to TB. All the women who died were newly diagnosed with HIV upon hospital admission, were severely immune suppressed, and had received a very short period of ART, if any, prior to delivery. They all had a short antituberculosis treatment duration (between 1 day and 1 month), making immune reconstitution inflammatory syndrome (IRIS) unlikely. Drug-resistant TB was confirmed in 6/42 (14%) women in whom *M*.*tuberculosis* was bacteriologically confirmed. We describe the drug-resistant TB from our cohort due to the lack of published data in this subgroup of TB patients. Noteworthy is that none of these women routinely received antituberculosis injectable agents during pregnancy, and that unfavourable maternal TB treatment outcomes were documented in four of these women.

Two-thirds of infants born to pregnant women with TB were premature and had LBW, exceeding the already high burden of LBW infant deliveries of 37% at our provincial referral hospital during the study period [12]. An increased risk of prematurity and LBW infant deliveries have previously been described in pregnant women with TB [10, 29]. Previous literature has also reported a high mortality in infants born to mothers affected by TB and HIV [4], all stillbirths and newborn deaths in our cohort were born to HIV-infected mothers. Congenital TB was only confirmed in 2/72 (3%) newborns, both in infants born to HIV-infected women, a lower figure than the 16% reported by Pillay et al., between 1997–1999, at a referral hospital in Durban, South Africa, prior to any ART use in HIV-infected pregnant women [1].

TB treatment completion or cure was only achieved in 41 (55%) women. These poor treatment outcomes are of concern and should be seen in the context of an academic hospital setting with more ill women referred for complications during pregnancy or birth. Almost a fifth of women with TB were lost to follow-up before being documented to continue treatment at the local TB clinic services (initial loss to follow-up), emphasizing the need to improve linking of clinical services between hospital and community-based TB clinics. Maternal HIV infection was not a predictor for an unfavourable TB treatment outcome; however, LBW deliveries were associated with unfavourable maternal TB treatment outcomes. It is possible that women with LBW infants may have had additional challenges to attend personal healthcare, given the challenges of also caring for these LBW infants.

This study has several limitations and does not represent the general population of pregnant women with TB in the study setting. Selection bias might have occurred as only women with complicated pregnancies are referred to TBH for specialised care. Despite this limitation, this relatively large cohort study of seventy-four women adds to the current limited knowledge base of TB disease during pregnancy and maternal TB treatment outcomes in a setting with high HIV prevalence. Another limitation was that the exact time of HIV diagnosis was not systematically recorded in all HIV-infected women. However, all women were tested for HIV prior to hospital discharge and all ART provision was recorded. TB and HIV treatment were provided as part of routine national programmatic services and antituberculosis treatment adherence was not monitored in this study. Follow-up was conducted by routine TB services, and only maternal TB treatment outcome data were collected by the study team. Our definition of unfavourable maternal TB treatment outcomes included women who were LTFU, as recommended by WHO. Although we cannot confirm that these women did not complete antituberculosis treatment elsewhere or whether they died, the use of two different TB registry documents (paper based and electronic) could not trace them.

In settings with high burden of TB and HIV, successful implementation of PMTCT programmes in HIV-infected women, with earlier initiation of ART, is critical to reduce maternal and infant morbidity and mortality. In addition, preventing TB deaths among HIV-infected women requires intensified scale-up of TB prevention, diagnosis and treatment interventions within maternal health and TB programmes. Basic antenatal TB screening should routinely be included in high burden TB/HIV settings, and the high risk of TB in the postpartum period should also be considered in TB screening guidelines [26]. Ascertaining previous TB treatment episodes may improve the sensitivity of the current TB symptom-screening tool. The use of improved molecular diagnostics, including Xpert MTB/RIF, which was not routinely available at the time of the study, may reduce diagnostic delays and result in more rapid initiation of antituberculosis treatment, also in pregnant women. A high degree of clinical awareness is essential to diagnose TB in pregnancy and the postpartum period. Improved recording and reporting of maternal TB and treatment outcomes during pregnancy will contribute to better estimates of disease burden, and inform much needed guidelines to improve TB case finding and outcomes amongst women and their infants.

## Supporting Information

S1 Dataset (XLSX)

## References

[1] Pillay T, Sturm AW, Khan M, Adhikari M, Moodley J, Connolly C, et al Vertical transmission of Mycobacterium tuberculosis in KwaZulu Natal: impact of HIV-1 co-infection. Int J Tuberc Lung Dis 2004;8:59–69. 14974747

[2] Khan M, Pillay T, Moodley JM, Connolly CA, Durban Perinatal TB HIV-1 Study Group. Maternal mortality associated with tuberculosis-HIV-1 co-infection in Durban, South Africa. AIDS 2001;15:1857–63. 11579249 10.1097/00002030-200109280-00016

[3] Bekker A, Du Preez K, Schaaf HS, Cotton MF, Hesseling AC. High tuberculosis exposure among neonates in a high tuberculosis and human immunodeficiency virus burden setting. Int J Tuberc Lung Dis 2012;16:1040–6. 10.5588/ijtld.11.0821 22691968

[4] Gupta A, Nayak U, Ram M, Bhosale R, Patil S, Basavraj A, et al Postpartum tuberculosis incidence and mortality among HIV-infected women and their infants in Pune, India, 2002–2005. Clin Infect Dis 2007;45:241–9. 10.1086/518974 17578786

[5] World Health Organization. Global Tuberculosis Report 2015. WHO, Geneva, Switzerland WHO/HTM/TB/2015.22. 2015.

[6] Deluca A, Chaisson RE, Martinson NA. Intensified case finding for tuberculosis in prevention of mother-to-child transmission programs: a simple and potentially vital addition for maternal and child health. J Acquir Immune Defic Syndr 2009;50:196–9. 10.1097/QAI.0b013e3181900201 19131888 PMC2884102

[7] World Health Organization. Global tuberculosis control 2008: surveillance, planning, financing, WHO, Geneva, Switzerland WHO/HTM/TB/2008.393. 2008.

[8] Mathad JS, Gupta A. Tuberculosis in pregnant and postpartum women: epidemiology, management, and research gaps. Clin Infect Dis 2012;55:1532–49. 10.1093/cid/cis732 22942202 PMC3491857

[9] Department of Health. National committee on confidential enquiries into maternal deaths. Saving mothers 2011–2013: sixth report on the confidential enquiries into maternal deaths in South Africa DOH, Pretoria, South Africa, 2015 [Accessed 29 July 2016]. Available from URL: www.kznhealth.gov.za/mcwh/Maternal/Saving-Mothers-2011-2013-short-report.pdf

[10] Jana N, Vasishta K, Jindal SK, Khunnu B, Ghosh K. Perinatal outcome in pregnancies complicated by pulmonary tuberculosis. Int J Gynaecol Obstet 1994;44:119–24. 7911094 10.1016/0020-7292(94)90064-7

[11] Bates M, Ahmed Y, Kapata N, Maeurer M, Mwaba P, Zumla A. Perspectives on tuberculosis in pregnancy. Int J Infect Dis. 2015;32:124–7. 10.1016/j.ijid.2014.12.014 25809768

[12] Western Cape Government. Annual Report Tygerberg Hospital 2012. Cape Town, South Africa, 2013. [Accessed 29 July 2016.] Available from URL: https://www.westerncape.gov.za/documents/annual_reports/2012.

[13] Wood R, Lawn SD, Caldwell J, Kaplan R, Middelkoop K, Bekker LG. Burden of new and recurrent tuberculosis in a major South African city stratified by age and HIV-status. PLoS One. 2011;6:e25098 10.1371/journal.pone.0025098 22016763 PMC3189963

[14] Department of Health. South African national tuberculosis management guidelines DOH, Pretoria, South Africa, 2009 [Accessed 29 July 2016.] Available from URL: http://www.tbonline.info/guidelines/

[15] World Health Organization. Definitions and reporting framework for tuberculosis—2013 revision WHO, Geneva, Switzerland WHO/HTM/TB/2013.2. 2013.

[16] World Heath Organization. Treatment of tuberculosis guidelines Fourth edition WHO, Geneva, Switzerland WHO/HTM/TB/2009.420. 2009.

[17] Bekker A, Slogrove AL, Schaaf HS, Du Preez K, Hesseling AC. Determinants of tuberculosis treatment completion among newborns in a high-burden setting. Int J Tuberc Lung Dis 2014;18:335–40. 10.5588/ijtld.13.0506 24670572

[18] Barron P, Pillay Y, Doherty T, Sherman G, Jackson D, Bhardwaj S, et al Eliminating mother-to-child HIV transmission in South Africa. Bull World Health Organ 2013;91:70–4. 10.2471/BLT.12.106807 23397353 PMC3537246

[19] Department of Health: Clinical Guidelines: PMTCT (Prevention of Mother-to- Child Transmission), 2010. [Accessed 21 May 2016.] Available at: http://www.fidssa.co.za/Guidelines

[20] World Health Organization. International statistical classification of diseases and related health problems, 10th revision, 1992 WHO, Geneva, Switzerland, 1992.

[21] Kriel M, Lotz JW, Kidd M, Walzl G. Evaluation of a radiological severity score to predict treatment outcome in adults with pulmonary tuberculosis. Int J Tuberc Lung Dis 2015;19:1354–60. 10.5588/ijtld.15.0098 26467588

[22] Ralph AP, Ardian M, Wiguna A, Maguire GP, Becker NG, Drogumuller G, et al A simple, valid, numerical score for grading chest x-ray severity in adult smear-positive pulmonary tuberculosis. Thorax. 2010;65:863–9. 10.1136/thx.2010.136242 20861290

[23] Doveren RF, Block R. Tuberculosis and pregnancy—a provincial study (1990–1996). Neth J Med 1998;52:100–6. 9599966 10.1016/s0300-2977(98)00004-7

[24] Hoffmann CJ, Variava E, Rakgokong M, Masonoke K, van der Watt M, Chaisson RE, et al High prevalence of pulmonary tuberculosis but low sensitivity of symptom screening among HIV-infected pregnant women in South Africa. PLoS One 2013;8:e62211 10.1371/journal.pone.0062211 23614037 PMC3629105

[25] Gupta A, Chandrasekhar A, Gupte N, Patil S, Bhosale R, Sambarey P, et al Symptom screening among HIV-infected pregnant women is acceptable and has high negative predictive value for active tuberculosis. Clin Infect Dis 2011;53:1015–8. 10.1093/cid/cir605 21940417 PMC3193828

[26] Zenner D, Kruijshaar ME, Andrews N, Abubakar I. Risk of tuberculosis in pregnancy: a national, primary care-based cohort and self-controlled case series study. Am J Respir Crit Care Med 2012;185:779–84. 10.1164/rccm.201106-1083OC 22161161

[27] Wong EB, Omar T, Setlhako GJ, Osih R, Feldman C, Murdoch DM, et al Causes of death on antiretroviral therapy: a post-mortem study from South Africa. PLoS One 2012;7:e47542 10.1371/journal.pone.0047542 23094059 PMC3472995

[28] Bates M, Mudenda V, Shibemba A, Kaluwaji J, Tembo J, Kabwe M, et al Burden of tuberculosis at post mortem in inpatients at a tertiary referral centre in sub-Saharan Africa: a prospective descriptive autopsy study. Lancet Infect Dis 2015;15:544–51. 10.1016/S1473-3099(15)70058-7 25765217

[29] Figueroa-Damian R, Arredondo-Garcia JL. Neonatal outcome of children born to women with tuberculosis. Arch Med Res 2001;32:66–9. 11282183 10.1016/s0188-4409(00)00266-6

